# Generation of UCiPSC-derived neurospheres for cell therapy and its application

**DOI:** 10.1186/s13287-021-02238-4

**Published:** 2021-03-18

**Authors:** Shuai Li, Huifang Zhao, Xiaobo Han, Bin Ni, Lang He, Omar Mukama, Jean de Dieu Habimana, Zuoxian Lin, Rongqi Huang, Hualin Huang, Chao Tian, Feng Tang, Zhiyuan Li

**Affiliations:** 1 CAS Key Laboratory of Regenerative Biology, Guangdong Provincial Key Laboratory of Stem Cell and Regenerative Medicine, Guangzhou Institutes of Biomedicine and Health, Chinese Academy of Sciences, Guangzhou, 510530 China; 2 Guangzhou Regenerative Medicine and Health Guangdong Laboratory, Guangzhou, 510005 China; 3 GZMU-GIBH Joint School of Life Sciences, Guangzhou Medical University, Guangzhou, China; 4 University of Chinese Academy of Sciences, Beijing, 100049 China; 5 NHC Key Laboratory of Birth Defect for Research and Prevention, Hunan Provincial Maternal and Child Health Care Hospital, Changsha, 410008 Hunan China; 6 Department of Anatomy and Neurobiology, Xiangya School of Medicine, Central South University, Changsha, Hunan China

**Keywords:** Human induced pluripotent stem cells, hiPSC-derived human neural stem cells, Neurospheres, Transportation, Ambient temperature

## Abstract

**Background:**

Neural stem cell (NSC) therapy remains one of the most potential approaches for the treatment of neurological disorders. The discovery of human induced pluripotent stem cells (hiPSCs) and the establishment of hiPSC-derived human neural stem cells (hiNSCs) have revolutionized the technique of cell therapy. Meanwhile, it is often required that NSCs are stored and transported to a long distance for research or treatment purposes. Although high survival rates could be maintained, conventional methods for cell transportation (dry ice or liquid nitrogen) are inconvenient and expensive. Therefore, the establishment of a safe, affordable, and low-cost strategy to store and transport easily accessible hiPSCs and hiNSCs, with characteristics that match fetal hNSCs, is incredibly urgent.

**Methods:**

We reprogrammed human urinary cells to iPSCs using a non-integrating, virus-free technique and differentiated the iPSCs toward iNSCs/neurospheres and neurons, under Good Manufacturing Practice (GMP)-compatible conditions. The pluripotency of iPSCs and iNSCs was characterized by a series of classical methods (surface markers, karyotype analysis, and in vitro as well as in vivo differentiation capabilities, etc.).

**Results:**

Here, our results showed that we successfully generated hiNSCs/neurospheres from more available, non-invasive, and more acceptable urinary cells by a virus-free technique. Next, we demonstrated that the iNSCs differentiated into mature cerebral cortical neurons and neural networks. Interestingly, hiNSCs survived longer as neurospheres at ambient temperature (AT) than those cultured in a monolayer. Within 7 days approximately, the neural viability remained at > 80%, while hiNSCs cultured in a monolayer died almost immediately. Neurospheres exposed to AT that were placed under standard culture conditions (37 °C, 5% CO_2_) recovered their typical morphology, and retained their proliferation and differentiation abilities.

**Conclusions:**

In this study, we provided a simple method for the storage of NSCs as neurospheres at AT as an alternative method to more costly and inconvenient traditional methods of cryopreservation. This will enable hiNSCs to be transported over long distances at AT and facilitate the therapeutic application of NSCs as neurospheres without any further treatment.

**Supplementary Information:**

The online version contains supplementary material available at 10.1186/s13287-021-02238-4.

## Background

In regenerative medicine, the clinical use of stem cell products is expected to bring substantial benefits to patients suffering from various diseases. Limbal stem cells have been registered as a product for eye burns in Europe [1]. Neural stem cells (NSCs) are multipotent cells that differentiate into the neurons and glia of the central nervous system. Due to their ability to self-renew and differentiate into the nervous tissue, they offer significant therapeutic potential in the treatment of neurological diseases such as spinal cord injury (SCI) [2], Alzheimer’s disease (AD) [3], and multiple sclerosis (MS) [4]. However, many currently available cell lines present us with severe obstacles relating to donor tissue acquisition, heterogeneity, availability, and related technical or ethical issues [5]. Besides, it is essential that cells must be transported from one place to another around the world for research and treatment and treatment under safe, stable, and affordable means that ensure cell survival for long distances and periods.

So far, many types of human cells have been reprogrammed to iPSCs, including skin fibroblasts [6], peripheral blood [7], periodontal ligament [8], keratinocytes [9], and adipose stem cells [10]. Compared to these, Zhou et al. discovered that urinary cells provide us a non-invasive, practical, and unlimited source for reprogramming [11]. Since the discovery of induced pluripotent stem cells (iPSCs) [6], induced neural stem cells (iNSCs) have rapidly progressed toward applications in neurodegenerative disease [12]. The availability of patient-specific iPSC-derived NSCs may alleviate difficulties of immunological issues and ethical concerns. For the systematic clinical use of iNSCs, the wide availability of donor cells would first be required. Furthermore, the properties of cellular behavior and the therapeutic efficacy of any iNSC preparations must be reproducible. To achieve this, complete stem cell functional characteristics need to be expressed and stabilized despite the extensive self-renewal of cells.

We have established a method in our laboratory to isolate cells from urine, reprogram them into iPSCs, then induce their differentiation into NSCs. This non-invasive method efficiently produces NSCs for use as a cell culture model to simulate neurological disease. However, human stem cells are susceptible to adverse external conditions, and their transportation relies on expensive and inconvenient cryopreservation. In this study, we showed that iNSCs survive longer as spheroids at AT than those cultured in a monolayer. We further researched and developed a novel method of NSC preservation that is time-saving and less labor-intensive in comparison with current storage methods, which is conducive to long-distance transportation of cells.

## Materials and methods

### Urinary cells collection and expansion

Zhou et al. detailed the procedure for urinary cell isolation and expansion in 2012 [11]. Typically, approximately 200 mL of urine were collected into sterile 50 mL tubes and centrifuged at 800*g* for 5 min at room temperature, followed by gently and quickly pouring out the supernatant, so as not to pour out the precipitate. About 5 mL of each tube is left and mixed into a centrifugal tube. Then, 20 mL of washing buffer was added and centrifuged at 800 g for 5 min at room temperature. The supernatant was carefully removed, leaving ~ 0.2 mL plus the pellet. Afterward, 1 mL of the primary culture medium was added to re-suspend the cell pellet, and then transferred the volume into a single well of a 12-well plate (coated beforehand with 0.1% gelatin). Next, 1 mL of the primary culture medium for the first 3 days was added, however without removing any medium. Approximately 4 days after plating, most of the medium was discarded, and 1 mL of REGM medium (LONZA, CC-3190, Switzerland) was subsequently added. Later, half of the medium could be changed every day to monitor the cell growth with a microscope until the density reached 80–90%. Finally, we split the obtained urinary cells (UCs) into ratios of 1:3 or 1:4 to a 6-well plate to allow them to expand quickly.

### hiPSC derivation and maintenance

The iPSCs were acquired by reprogramming the UCs as described in the previous work [11]. The iPSCs were reprogrammed from the UCs of a healthy 20–30-year-old person using the same method. For the following experiments, all iPSCs were cultured on 5 μL/mL Matrigel (Corning, 354277, USA) in mTeSR™1 (Stemcell Technologies, 5872, Canada).

### Generation and differentiation of iPSC-derived NSCs through TGF-β/Smad and BMP inhibition

hiPSCs were expanded for 4 days in mTeSR™ 1 medium. Undifferentiated hiPSC colonies were broken into fragments using a P1000 pipette and re-plated onto 5 μL/mL Matrigel-coated dishes in embryoid bodies (EBs) + 2i medium (DMEM/F12, 20% KSR, NEAA (1×), GlutaMax (1×), 0.1% beta-Mecaptoenthano, 5 μM SB431542, and 5 μM dorsomorphin) to generate EBs for 4 days. The EBs were re-plated onto Matrigel-coated dishes in N2B27 medium (1:1 mixture of N2 and B27; N2 medium consists of 1 × N2, DMEM/F-12, NEAA (1×), GlutaMAX (1×), 5 μg/mL insulin, 1 mM L-glutamine, 100 μM 2-mercaptoethanol; B27 medium consists of Neurobasal, 1 × B27). Neural rosettes were visible and matured within 14 days. Rosettes were picked and then dissociated into single cells in accutase for 2 min (Sigma, A6964, USA) that are suspended in culture. After 7–10 days, the single cells produced round neurospheres in N2B27 medium. Neurospheres were collected in a 15 mL tube with N2B27 medium and could be stored at AT for a week. To further acquire neurons, neurospheres were dissociated into single cell using accutase and transferred onto Matrigel-coated 24-well plate at the density of 2 × 10^4^ cells/well in N2B27 medium and then change the culture medium every other day. Cells were cultured for 1 month and obtained a mixture of neural cells containing astrocytes and neurons was obtained.

### Karyotype analysis

Karyotype analysis was performed in iPSCs at passage 15 and in iNSCs at passage 5. When the cells had reached the logarithmic phase, Colcemid was added to a final concentration of 20 μg/mL for 2 h. The supernatant was removed, and the pellet was resuspended in 8 mL of 0.075 M KCl and incubated at 37 °C for 20 min. The cells were fixed with fresh Carnoy’s Fixative (3:1 ratio of methanol: glacial acetic acid). Twenty metaphases were analyzed at 450–500 band resolution using Ikaros (MetaStstems, Germany) on an Olympus BX51 microscope.

### Teratoma formation

Human iPSC cells were harvested in 1.5 mL tube and two million cells were injected into the flank subcutaneously and Lower limb intramuscularly of NOD/SCID mice. After 8–10 weeks, euthanasia of mice with rising CO_2_ levels was performed. Tumors were collected and embedded in paraffin, stained with hematoxilin/eosin, and then histologically analyzed.

### Immunofluorescence staining

Cells were briefly fixed in 4% paraformaldehyde for 15 min at room temperature. After permeabilization with 0.5% Triton X-100 in PBS for 5 min, the cells were blocked with 0.5% Triton X-100 and 10% goat serum for 30 min. Next, the cells were incubated with primary antibodies in 10% goat serum at 4 °C overnight and then incubated with secondary antibodies (Supplementary Table 2) diluted in 10% goat serum for 1 h at room temperature. Nuclei were counterstained with DAPI (Beyotime Biotechnology, C1005, China) for 5 min. Images were acquired with an inverted fluorescence microscope (Olympus, IX73, Japan).

### Quantitative real-time PCR (qRT-PCR)

Total RNA was extracted using TRIzol reagent (Invitrogen, 15596026, USA). Total cDNA was prepared with HiScript II Q RT SuperMix for RT-qPCR (Vazyme, R223-01, China). qRT-PCR was then performed using specific primers in a CFX96 Real-Time System (Bio-Rad, USA). Primers are listed in Supplementary Table 2.

### Flow cytometry

Cells were collected and fixed in 4% paraformaldehyde for 30 min at 37 °C. After washing with PBS, the cells were resuspended in FACS buffer. Then, the cells were incubated with the primary antibodies overnight at 4 °C, and secondary antibodies were carried out at room temperature for 30 min (Table 2). After washing, the cells were resuspended in 200–300 μL of the FACS buffer and proceeded for analysis on BD Accuri C6 Plus. The data were analyzed with FlowJo V10 (FlowJo LLC, BD) and BD Accuri C6 Plus software.

### Electrophysiology

Electrophysiological recordings were performed at using a whole-cell, voltage- or current-clamp technique. Whole-cell recordings were made with 6–9-MΩ borosilicate glass electrodes and specific protocols were depicted in each figure. An Axopatch 200B amplifier (Axon Instruments, USA) was used to record the electrophysiological signals. The data were acquired and analyzed using Clampfit 10.2 software (Molecular Devices, USA). Borosilicate glass pipettes had resistances of 4–8 MV when filled with a solution containing the following (mM): 140 potassium methanesulfonate, 10 HEPES, 5 NaCl, 1 CaCl_2_, 0.2 EGTA, 3 ATP-Na_2_, 0.4 GTP-Na_2_, pH 7.2 (adjusted with KOH). The bath solution contained the following (mM): 127 NaCl, 3 KCl, 1 MgSO4, 26 NaHCO3, 1.25 NaH_2_PO_4_, 10 D-glucose, 2CaCl_2_, pH 7.4 (adjusted with NaOH). Cells plated on coverslips were placed in a submerged recording chamber and were continually perfused with the bath solution equilibrated with 95% O_2_ and 5% CO_2_. All electrophysiological experiments were performed at room temperature.

### Mycoplasma test

The Lonza MycoAlert™ mycoplasma detection kit was used to estimate the mycoplasma according to the instruction.

### Short tandem repeat (STR) analysis

STR analysis was performed on the urine cells and established iPSCs with detection of 21 loci (Amelogenin, D3S1358, vWA, D7S820, CSF1PO, PentaE, D8S1179, D21S11, D16S539, D2S1338, PentaD, D19S433, TH01, D13S317, THOX, D18S51, D6S1043, D1S1656, D5S818, D12S391, FGA) by GUANGZHOU IGE BIOTECHNOLOGY LTD, China.

### Data analysis

Statistical analysis for all the experimental data was performed using GraphPad Prism 7 and Microsoft Excel. The data were presented as mean ± SD. Statistical significance was determined using paired *T* test.

## Results

### Generation and characterization of UC-derived hiPSCs

**Fig. 1:**
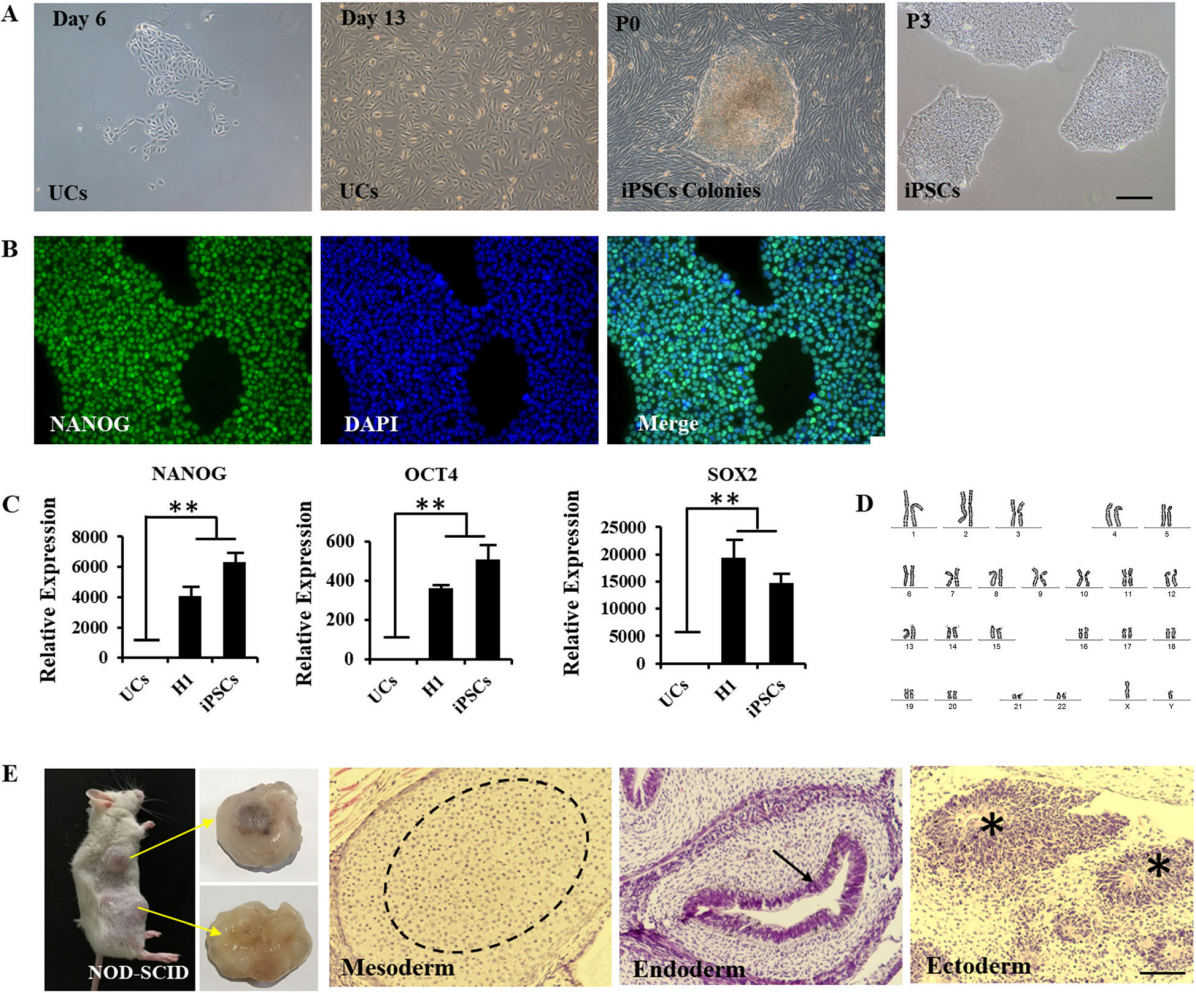
Characterization of iPSCs lines obtained upon reprogramming of urinary cells from normal donor. **a** Morphology from urinary cells to iPSCs at different time points. **b** Immunofluorescent labeling for pluripotency markers NANOG. **c** qRT-PCR assay for expression of endogenous human pluripotency genes in this iPSCs lines, with UCs as negative control and H1 as positive control (*n* = 3). **d** G-band analysis of the iPSCs showed a normal karyotype. **e** Teratoma formation of iPSCs lines. iPSCs injection into NOD-SCID immunodeficient mice resulted in teratoma formation (Left, *n* = 6). HE-staining showed that the capacity for iPSCs differentiation into three germ layers was certified by teratoma formation in vivo (right): mesoderm (cartilage, circle), endoderm (gut-like epithelium, arrow), and ectoderm (neural rosette, asterisk). **indicates *P* < 0.01. Scale bar: 100 μm (**a**); 50 μm (**b**, **e**)

With a consent form signed by the donor, we collected and established a normal human UC in about 13 days. We then constructed an episomal vector expressing human miR-302-367 cluster and transfected it into UCs with OCT4, SOX2, SV40T, KLF4 (OSTK) using electroporation (Supplementary Fig. 1). The transfected UCs were cultured on Matrigel-coated and serum-free medium (mTesR1) for reprogramming. The iPSCs showed a typical human embryonic stem cell (hESC) morphology with a high nuclear to cytoplasmic ratio around 20 days after electroporation (Fig. 1a) and expressed specific marker NANOG (Fig. 1b). Real-time polymerase chain reaction (RT-qPCR) results displayed that the endogenous pluripotency genes (NANOG, OCT4, and SOX2) were at a high expression rate in hiPSCs with parental UCs and comparable level with human embryonic stem cell (H1) (Fig. 1c). G-band analysis of the iPSCs (> 15 passages) showed normal diploid 46, XY karyotype (Fig. 1d). The NOD-SCID mice were injected with hiPSCs in the underarm skin and hindleg muscles and the capacity for differentiation into three germ layers was certified by teratoma formation in vivo, such as cartilage (mesoderm), gut-like epithelium (endoderm), and neural rosette (ectoderm) (Fig. 1e). This hiPSCs were negative for mycoplasma test (Supplementary Table 1). Finally, STR analysis also verified the genetic identity of the cell line and the parental urine cells (Supplementary Table 3). In conclusion, we generated a non-invasive, virus-free UC-derived iPSCs (named C1P4) from human UCs under feeder-free and xeno-free conditions.

### Generation and characterization of iPSC-derived NSCs

**Fig. 2:**
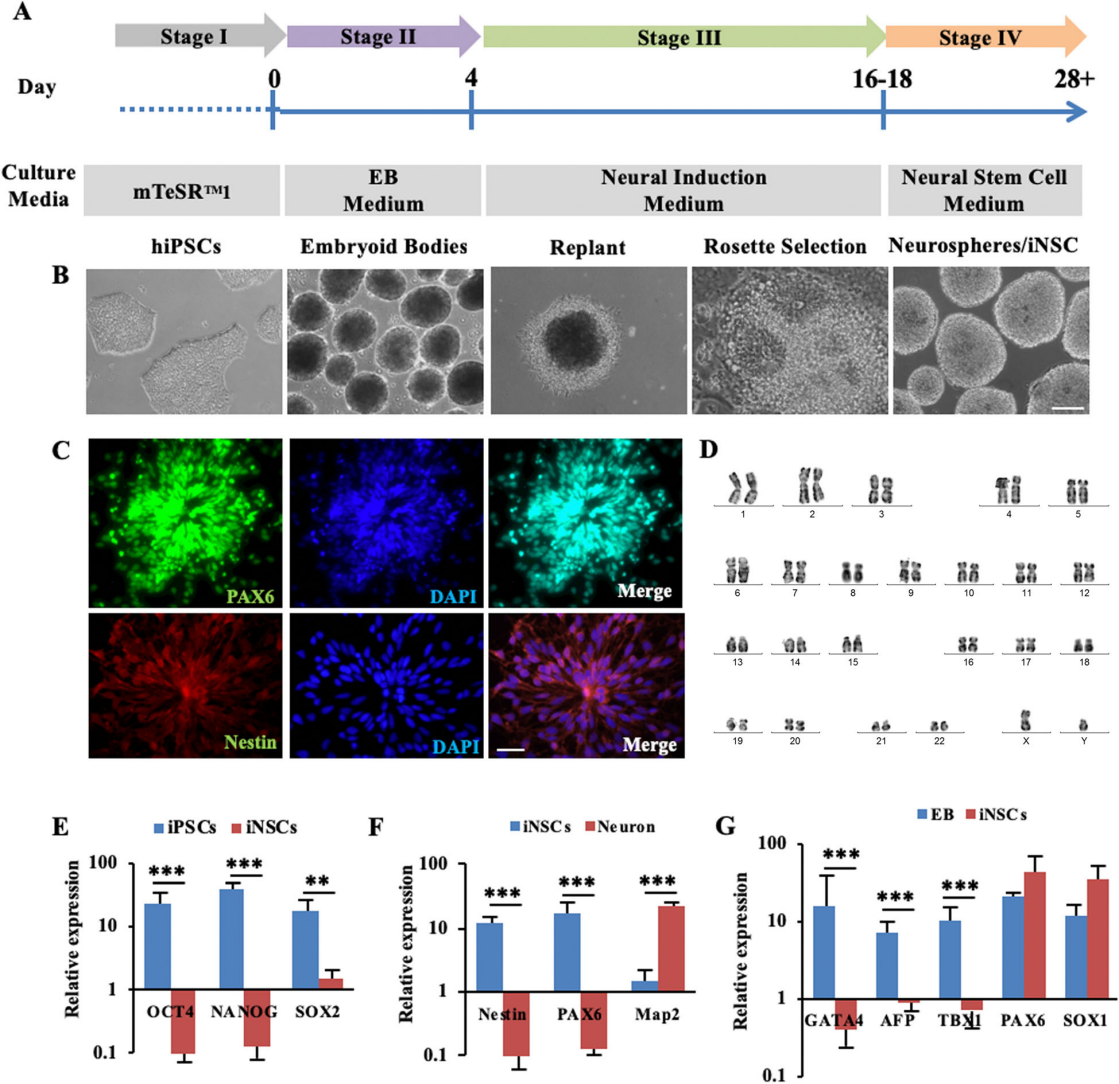
Generation of induced neural stem cells (iNSCs) from hiPSCs via single BMP inhibition. **a**, **b** Protocol timeline and representative images to describe the different stages of generating Neurospheres/iNSCs from hiPSCs. **c** Immunofluorescence staining with NSC markers PAX6 and Nestin. **d** G-band analysis of the iNSCs shows normal karyotype. **e** Bar graphs of qRT-PCR showing mRNA-expression profile of iNSCs respect to iPSCs (*n* = 3). **f** Bar graphs of qRT-PCR showing mRNA-expression profile of iNSCs with respect to neuron (*n* = 3). **g** Bar graphs of qRT-PCR showing mRNA-expression profile of iNSCs with respect to EB (*n* = 3). **indicates *P* < 0.01, ***indicates *P* < 0.001. Scale bar: 100 μm (**b**); 50 μm (**c**)

**Fig. 3:**
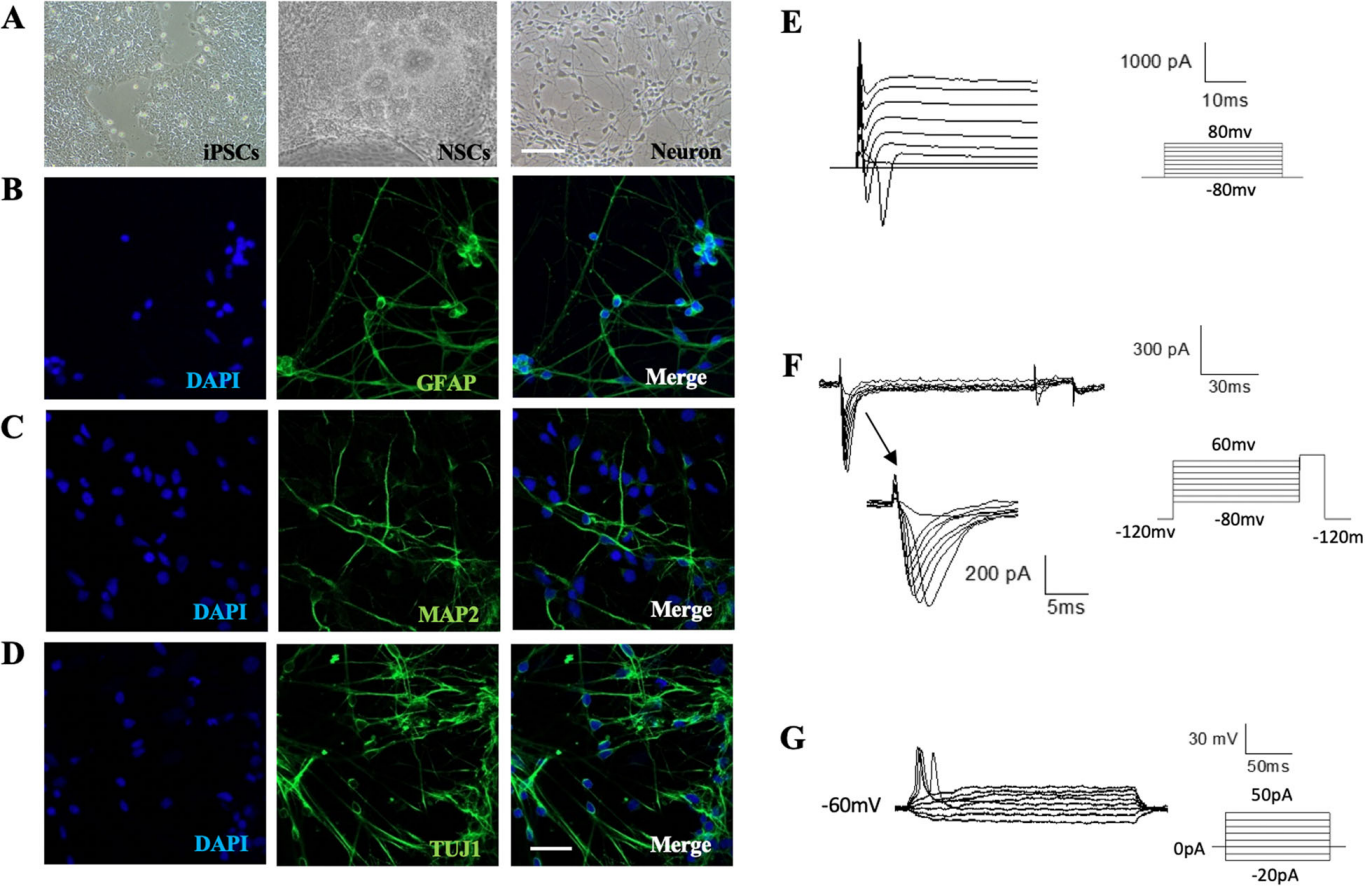
iNSCs differentiate into mature neurons. **a** Representative images of differentiation at different stages. **b**–**d** Immunofluorescence staining of differentiated neurons derived from iNSCs for mature cortical neuronal markers (TUJ1, MAP2), glial markers (GFAP). Nuclei stained with DAPI. **e** Representative traces of Na^+^-k^+^-Ca^2+^ currents, the neurons expressed strong currents. **f** The result shows the voltage-gated sodium currents, indicated by the arrow, recorded following depolarizing voltage steps (− 80 to 60 mV, *n* = 5). **g** Action potentials (AP) evoked in response to step current respectively (− 20 to 50 pA, *n* = 3). Scale bar: 100 μm (**a**); 50 μm (**b**–**d**)

We used a highly efficient method to induce NSCs (iNSCs) from iPSCs and their differentiation into neurons. The steps of the large-scale generation of iNSCs and neurons using embryoid bodies methods are schematically described in Fig. 2a. iPSCs are expended for 4 days in mTeSR™1 medium. Undifferentiated hiPSC colonies were broken into fragments using a P1000 pipette and re-plated onto Matrigel-free dishes in EBs medium to generate EBs for 4 days. The EBs were re-plated onto Matrigel-coated dishes. Neural rosettes were visible and matured within 14 days. Rosettes were picked and then dissociated into single cells with Accutase that are suspended in culture. After 7–10 days, the single cells produced round neurospheres (Fig. 2b). In order to determine the actual fate commitment of the generated neurospheres/iNSCs, we performed further differentiation into neurons (Fig. 3a).

To prove the presence of iNSCs, we performed immunocytochemical staining for NSC markers (PAX6, Nestin). The iNSCs showed positive PAX6, Nestin staining (Fig. 2c). Furthermore, the analysis of flow cytometry showed that the cells positively express 91.2% and 89.2% of the neuroectodermal marker PAX6 and Nestin, respectively (Supplementary Fig. 3A). G-band analysis of the iNSCs (> 5 passages) showed normal diploid 46, XY karyotype (Fig. 2d). Next, to exclude the permanence of cells expressing hiPSC markers in iNSC cultures, the expression of iPSC markers in iNSCs was compared to parental iPSCs. Unlike iNSCs, iPSCs expressed detectable levels of pluripotency markers (OCT4, NANOG, and SOX2) (Fig. 3e). In contrast, NSC-positive marker Nestin and PAX6 were expressed, but neuron-positive marker Map2 was not expressed by iNSCs (Fig. 3f). To preclude the presence of non-neuroectoderm cells, we compared the expression of mesodermal, endodermal, and neuroectodermal cell line markers of iNSC to EBs. iNSCs did not express GATA4, AFP (entoderm), and TBX1 (mesoderm), but maintained a high expression of PAX6 and SOX1 (ectoderm) (Fig. 3g). Therefore, we obtained a cell line expressing the properties of NSCs by the EBs method.

### iNSCs differentiate into cerebral cortical neurons

In order to confirm that the iNSCs have the characteristics of functional progenitors, we differentiated the iNSCs into mature cerebral cortical neurons and neural networks. We used a method in the previous protocol [13] and single-celled iNSCs at passage 5 were plated onto Matrigel-coated dishes in neural differentiation medium for 30 days before performing immunofluorescent staining. To identify the mature neurons obtained, we used neuron-specific cytoskeletal marker TUJ1, dendritic marker Map2, and astrocyte marker GFAP. The iNSCs successfully differentiated into cortical neuron and astrocyte, as confirmed by the presence of staining for the cortical neuron and astrocyte markers (Fig. 3b–d). Moreover, we performed flow cytometry and the results showed that 71% of the cells positively express neuron marker MAP2 and 21.8% of the cells express glial cells marker GFAP (Supplementary Fig. 3B). To further characterize the neurons, whole-cell patch-clamp recordings were performed to assay the functional maturity of the iNSC-derived neuronal networks. The neurons exhibited sodium, potassium, and calcium channel activities by generating inward voltage-gated sodium currents and outward voltage-gated potassium currents (Fig. 3e, f). The neurons had evoked action potentials after injection step currents (Fig. 3g).

### Neurospheres formation extends iNSCs survival under ambient temperature

**Fig. 4:**
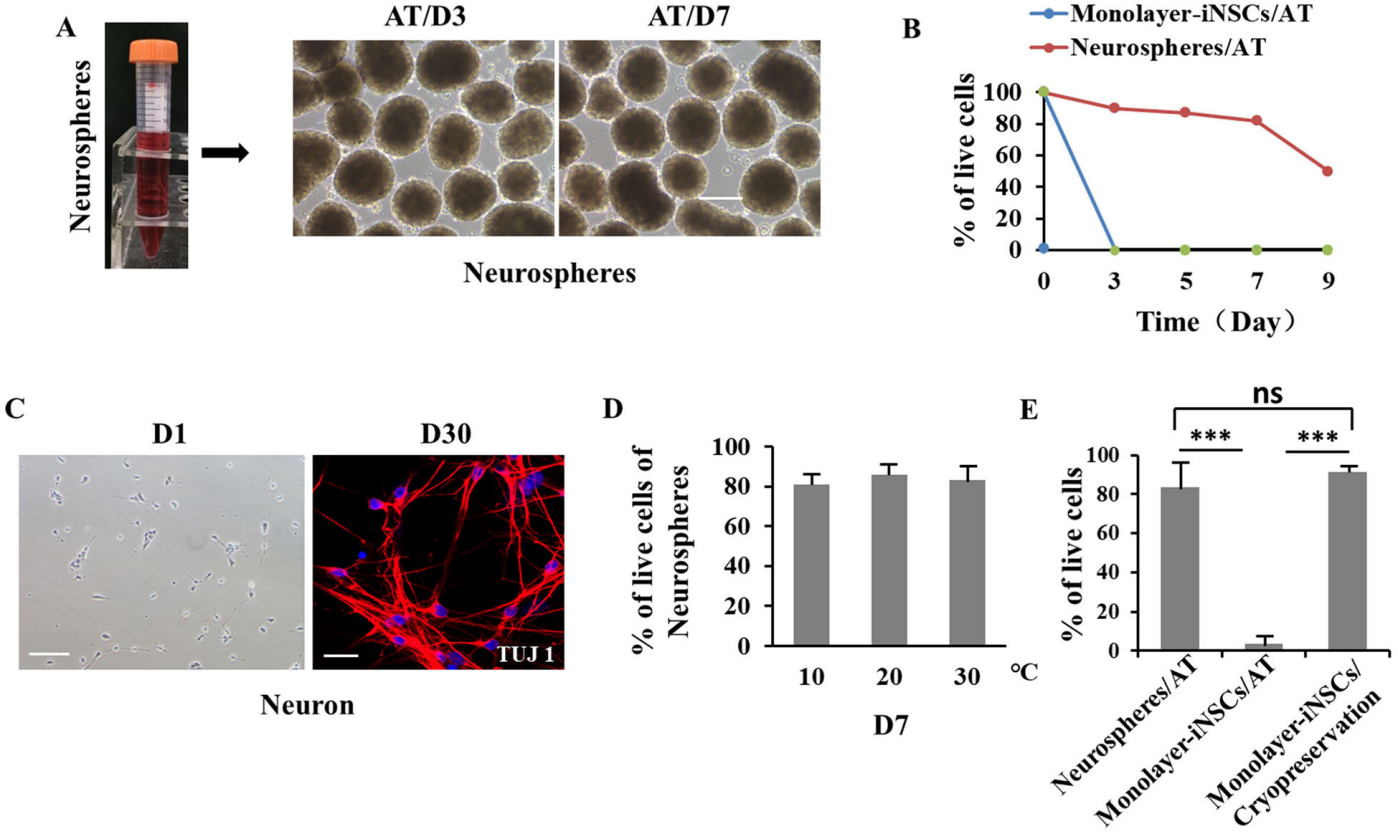
Neurospheres strengthen survival of AT-exposed iNSCs. **a** iNSC monolayer was cultured to form neurospheres and exposed to AT for D3 and D7. **b** The survival rates of monolayer-iNSCs/AT and neurospheres/AT were compared at different time points (*n* = 3). **c** The neurospheres/D7 were isolated and then differentiated into neurons. Neurons were stained using TUJ1. **d** The survival of neurospheres in 10, 20, and 30 °C, respectively. **d** Comparison of three methods of storing iNSCs (*n* = 3). ***indicates *P*  < 0.001, ns indicates no statistical significance. Scale bars: 100 μm (A: neurospheres; neuron/D1); 50 μm (A: neuron/D30)

In this study, we aimed to understand the effect of AT treatment on the viability of iNSCs and establish a simple approach for cell storage and shipment. Human iPSCs were induced to differentiate into neurospheres using the method we mentioned previously (Fig. 2). Neurospheres were then collected, put into a 15 mL centrifuge tube, and placed on the cell room under AT for 7 days (Fig. 4A). For comparison, iNSCs were cultured in monolayer on a 6-well plate under AT for 7 days as well. Consequently, the monolayer-iNSCs quickly floated up and died within 3 days. However, the neurospheres did not change significantly in morphology within 7 days under AT, and then the survival rate dropped rapidly on day 9 (Fig. 4b). To prove that the neurospheres still have the characteristics of functional progenitors after 7 days, we first performed the qRT-PCR and the result showed the neurospheres/day 7 still express positive markers (PAX6, Nestin) of NSCs (Supplementary Fig. 2). Meanwhile, we differentiated the neurospheres into mature cerebral cortical neurons. Immunofluorescence results showed that the neurospheres still differentiate into neurons (Fig. 4c). In order to test whether neurospheres can withstand temperatures other than AT, neurospheres were respectively stored at 10, 20, and 30 °C, and the survival rate of neurospheres basically unchanged (Fig. 4d). The survival rates of neurospheres/AT and monolayer-iNSCs/AT were compared with those of monolayer-iNSCs thawed after conventional cryopreservation. The result revealed that monolayer-iNSCs cannot tolerate AT for long periods of time. On the other hand, neurospheres/AT and monolayer-iNSCs/cryopreservation achieved similar survival rates (Fig. 4b, e).

## Discussion and conclusions

Nervous system diseases, such as spinal cord injury (SCI), autism spectrum disorder (ASD) are associated with the dysfunctional recovery and limited regenerative capacity of the central nervous system (CNS), which cannot repair or replace neurons and axons after injury. Transplantation of stem cells, especially neural stem cells (NSC), can repair or replace damaged neurons and glial cells by providing a suitable microenvironment and enhancing their regeneration. Substantial preclinical researches have furthered the use of Neural Precursor Cells in clinical trials [14–16]. A preclinical study showed that NSCs induced by iPSCs in healthy 86-year-old males still function in transplantation therapy [17].

iPSCs produced by reprogramming adult cells into a self-renewing pluripotent state eliminate the ethical issues associated with the use of human fetal/embryonic tissue and reduce immune rejection of implanted cells. However, the virus-based methods used in most reports caused security concerns, which limit their further application. Recently, several methods have been reported to generate safer iPSCs by using adenovirus, Sendai virus, expression plasmids, self-replicating RNA, episomal plasmid vectors. Adenovirus is a non-integrating virus, but the reprogramming efficiency is 0.0002% in human cells. Sendai virus is an RNA virus that does not enter the nucleus and can produce large amounts of protein. One shortcoming is that it takes ~ 10 passages for the virus to be completely lost from reprogrammed iPSCs [18]. Heidrun Steinle et al. have reported that footprint-free iPSCs were successfully generated through a single application of self-replicating RNA (srRNA) that encodes reprogramming factors (OCT4, KLF4, SOX2, and c-MYC) [19]. In here, we reprogrammed UCs using the previously reported episomal system without serum, feeder, and oncogene c-MYC. We used the previously constructed episomal vector expressing human miR-302-367 Cluster and transfected it into UCs with OSTK through electroporation [20]. We finally obtained the iPSCs colonies.

UCs are exfoliated epithelial cells of the kidney system and can be collected in any condition except renal failure [11]. Thus, urine shed cells provide us with a practical and unlimited source of human cells for reprogramming, and this non-invasive approach to human cell acquisition will significantly improve patient compliance. Besides, epithelial to mesenchymal transformation (EMT) is essential for somatic cells to become stem cells, and UCs, as a renal epithelial cell, is easier to overcome this transformation due to its epithelial origin [11]. Therefore, UCs are a very useful tool for studying cell therapy and tissue engineering.

Although numerous studies have demonstrated the clinical potential of iPSC-derived NSCs in treating neurological diseases, there are still some problems and challenges. The iPSC/iNSC generation method needs to be better optimized to produce cells with good therapeutic efficacy and minimal adverse side effects, so as to make them more valuable for clinical use. The clinical use of iNSCs depends mainly on the availability of a suitable donor cell source, which must remain reproducible and predictable enough to produce the desired number of cells. This large-scale production ensures uncompromised cellular therapy of patients. Furthermore, temperature is also one of the main influencing factors for the survival of cells in vitro and high viability after thawing is an urgent requirement for cell transportation [21].

Combination of dorsomorphin (BMP signals inhibitor) and SB431542 (TGFb/activin/nodal signals inhibitor) promoted neural induction [22]. In this study, by using this combination in the first step of the differentiation scheme, we discovered the formation of representative rosette structures of early-stage ES- or iPSC-derived neural precursors, and then generated and characterized a population of virus-free hiPSC-derived hiNSCs expressing typical NSC markers (PAX6 and Nestin) and further differentiated them into neurons that exhibit a characteristic feature. Like ES-derived iNSCs, our iNSCs had a normal karyotype and settled amplification efficiency, did not possess telomerase activity. Our iNSCs preparation demands 1 month, followed by another 1 month for expansion. Thus, our protocol can produce a large amount of stably iNSCs for several serial transplants from clinical-grade UC-derived iPSCs.

Cells need to be frozen and transported for any particular use. For long time storage, the traditional cell cryopreservation method requires − 80 °C refrigerator or liquid nitrogen long time. Moreover, conventional transportation methods require a particular container, including dry ice or liquid nitrogen, to keep low temperatures. Neural stem cells also need to be preserved like this. But with traditional preservation, it is very difficult to ensure cells survived at room temperature for a long time. Bin Jiang et al. found spheroidal formation preserves MSCs for prolonged time under ambient conditions for facile storage and transportation [23]. We demonstrated the feasibility of iNSCs as neurospheres storage and transportation at AT. This will remarkably facilitate long-distance transportation and therapeutic application of iNSCs without further processing. In our experiments, we found that neurosphere could be stored for about 7 days, and the survival rate could reach more than 80%. This method of transporting neural stem cells has the advantages of being simple, low cost, and does not require special containers or dry ice. The living cells can be quickly recovered and put into working state after being transported to the destination. Cell viability is also acceptable compared to traditional methods. Therefore, this neurosphere preservation method meets the cell transportation between cities and countries as well.

**Fig. 5:**
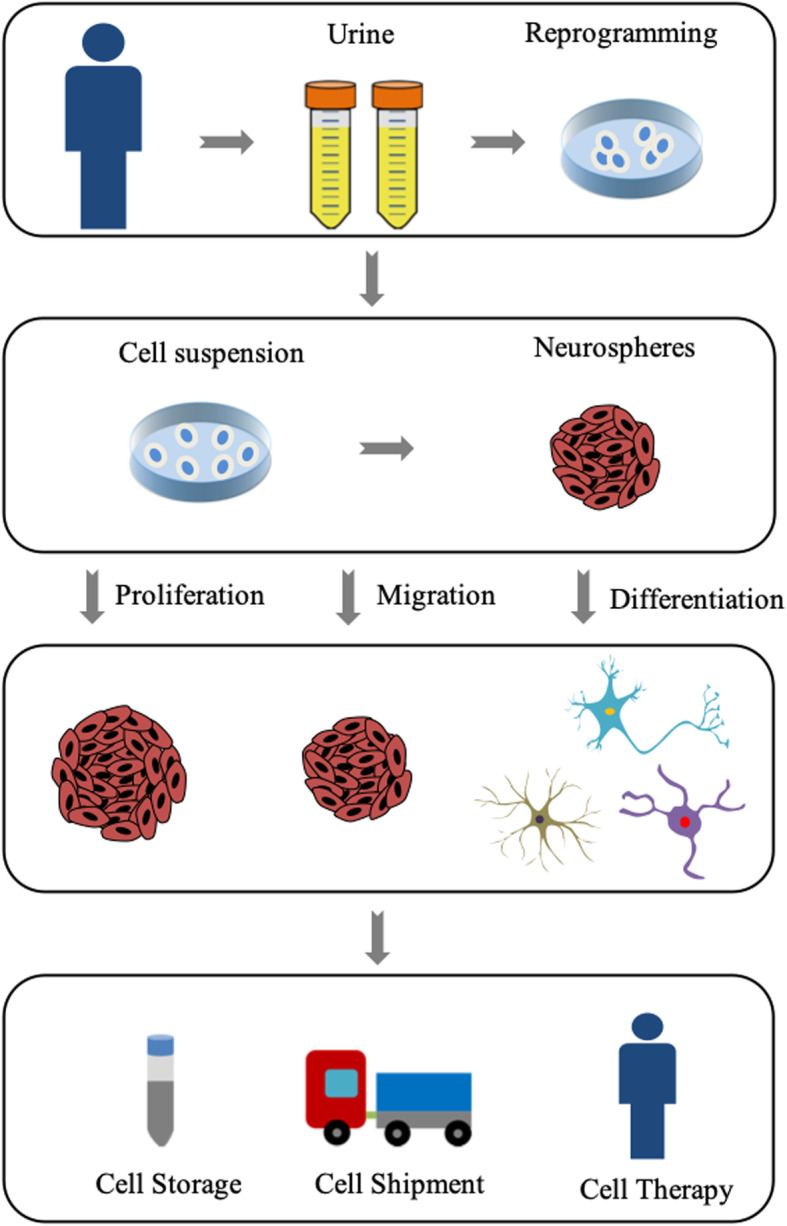
Model summarizes the whole process schematically from reprogramming to the application of neurospheres

In conclusion, generating iNSCs from UC-derived iPSCs and the formation of the neurosphere for transportation and storage as described here may have several distinct advantages. First, urine collection is simple and straight-forward compared with other sources of human biological material, which simplifies ethical issues. Second, the application of NSCs was commonly under 10 passages for maintaining relatively high stemness [24]. UC-derived iPSCs could guarantee that the number of iNSCs is almost unlimited for the treatment of neurological diseases. Third, the form of neurosphere is conducive to transportation and storage at AT, and it is easy to scale production. Figure 5 summarizes the whole process schematically from reprogramming to the application of neurospheres. However, there are still some problems. We still need a lot of animal experiments to prove its feasibility in vivo before it can be used in the clinical stage.

## Supplementary Information

**Additional file 1: Supplementary Fig. 1.** Schematic flow diagram to describe the stages of induction into iPSCs from Urinary cells.

**Additional file 2: Supplementary Fig. 2.** qRT-PCR assay for expression of NSCs genes in neurospheres/Day7, with iPSCs and neurons as negative control, and neurospheres/Day0 as positive controls.

**Additional file 3: Supplementary Fig. 3.** Flow cytometry assay.

**Additional file 4 Supplementary Table 1.** This iPSCs were negative for mycoplasma test.

**Additional file 5: Supplementary Table 2.** Antibodies used for immunocytochemistry.

Additional file 6: **Supplementary Table 3.** STR analysis also verified the genetic identity of the cell line and the parental urine cells.

## Data Availability

The data that support the findings of this study are available from the corresponding author upon reasonable request.

## Abbreviations

NSCs: Neural stem cells
hiPSCs: Human induced pluripotent stem cells
hiNSCs: hiPSC-derived human neural stem cells
SCI: Spinal cord injury
AD: Alzheimer’s disease
MS: Multiple sclerosis
UCs: Urinary cells
hESC: Human embryonic stem cell
EBs: Embryoid bodies
AT: Ambient temperature
ASD: Autism spectrum disorder
CNS: Central nervous system
