# Supplementary figures and images for: Generation of UCiPSC-derived neurospheres for cell therapy and its application
Source: Stem Cell Res Ther. 2021 Mar 18;12:188. doi: 10.1186/s13287-021-02238-4 (PMC7977190; doi:10.1186/s13287-021-02238-4)

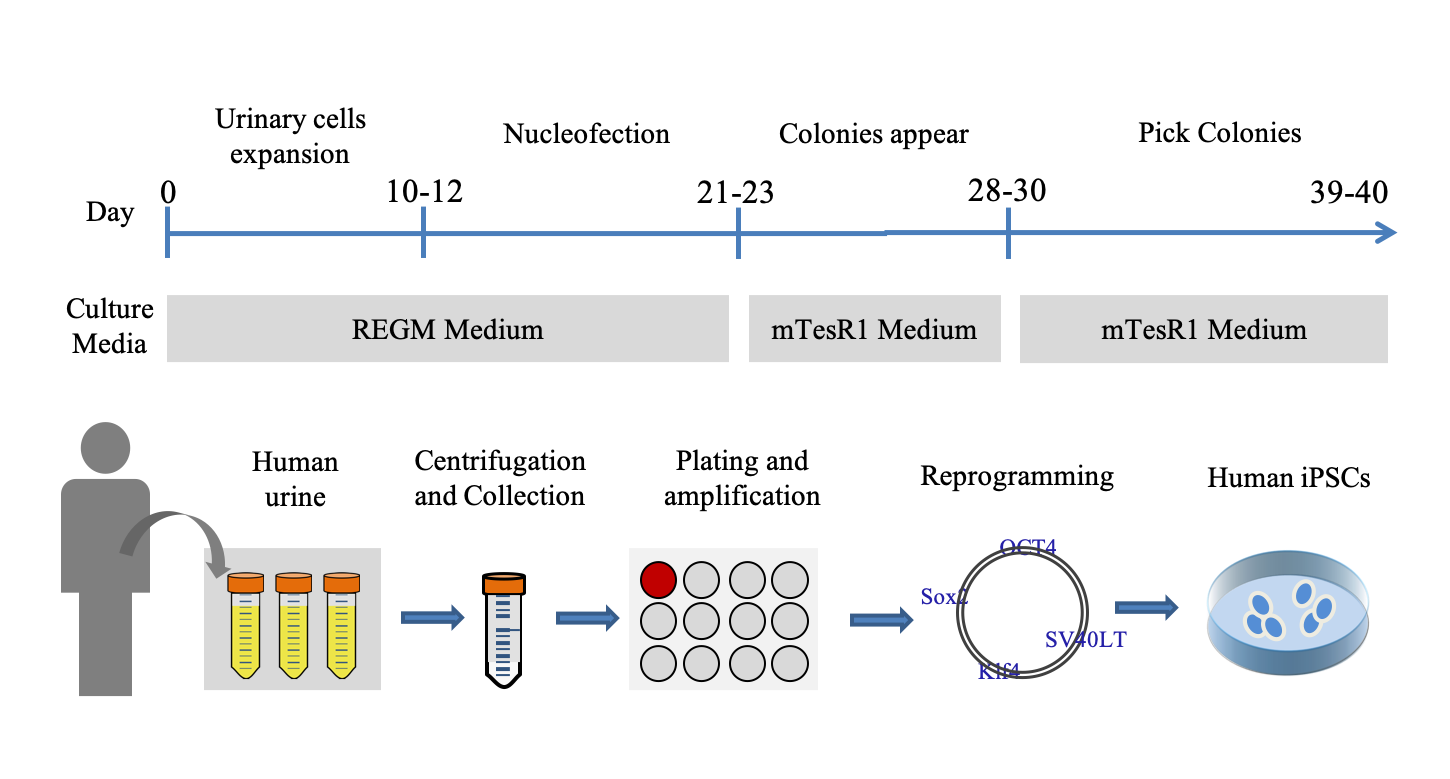

Supplement: Supplementary file 1 — Additional file 1: Supplementary Fig. 1. Schematic flow diagram to describe the stages of induction into iPSCs from Urinary cells. [file 13287_2021_2238_MOESM1_ESM.png]

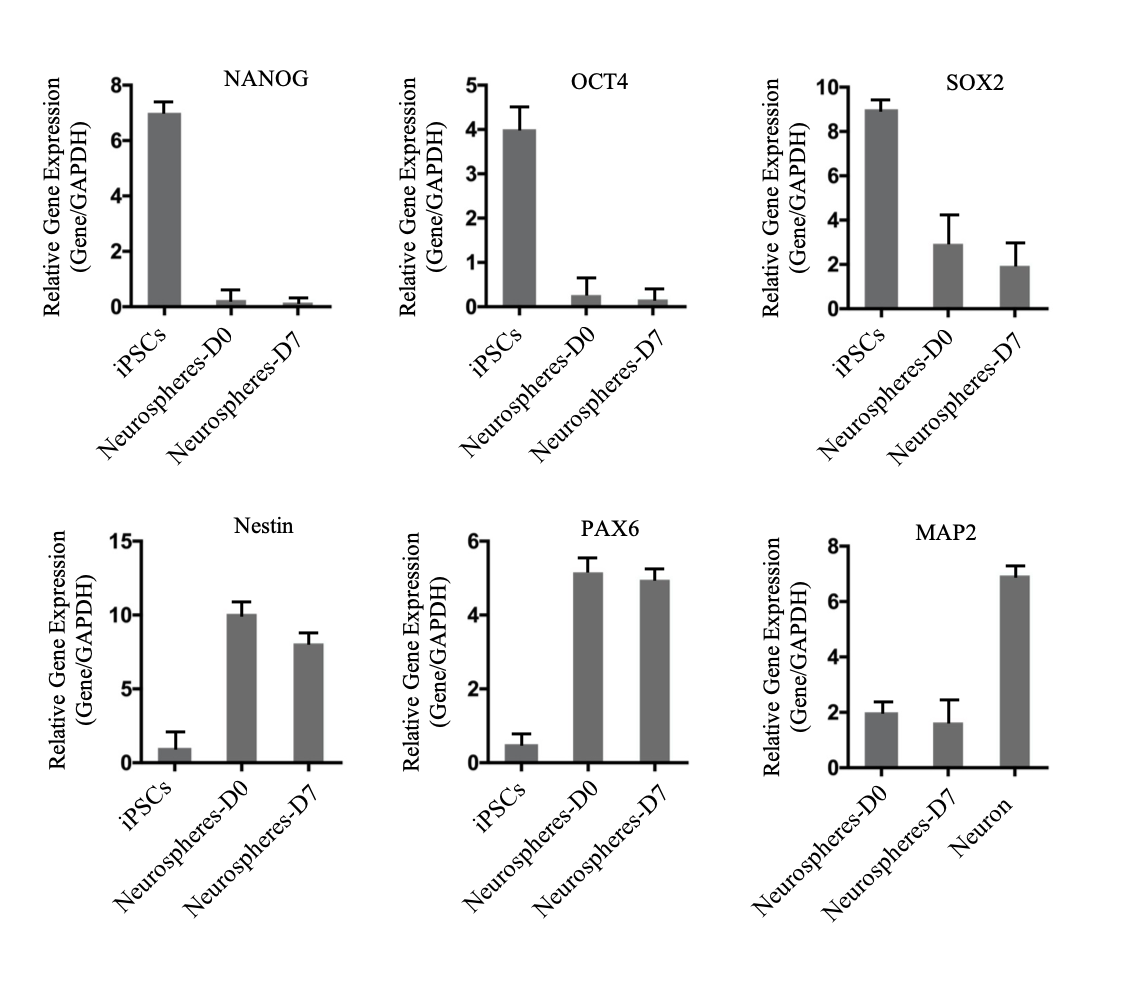

Supplement: Supplementary file 2 — Additional file 2: Supplementary Fig. 2. qRT-PCR assay for expression of NSCs genes in neurospheres/Day7, with iPSCs and neurons as negative control, and neurospheres/Day0 as positive controls. [file 13287_2021_2238_MOESM2_ESM.png]

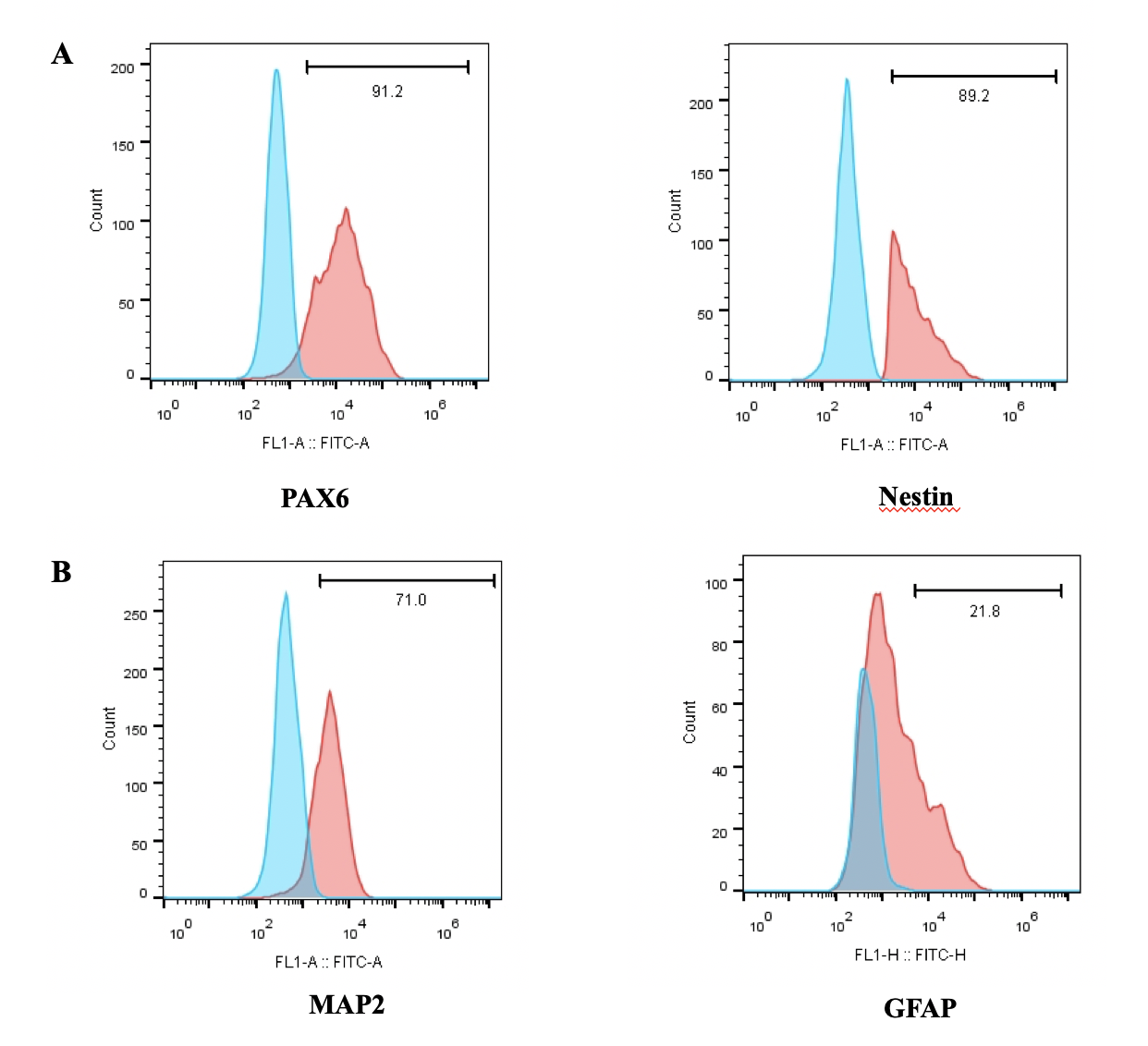

Supplement: Supplementary file 3 — Additional file 3: Supplementary Fig. 3. Flow cytometry assay. [file 13287_2021_2238_MOESM3_ESM.png]
