# Supplementary material for: Generation of UCiPSC-derived neurospheres for cell therapy and its application
Source: Stem Cell Res Ther. 2021 Mar 18;12:188. doi: 10.1186/s13287-021-02238-4 (PMC7977190; doi:10.1186/s13287-021-02238-4)
Supplement: Supplementary file 4 — Additional file 4 Supplementary Table 1. This iPSCs were negative for mycoplasma test. [file 13287_2021_2238_MOESM4_ESM.docx]

**Supplementary Table 1: Mycoplasma Test**

| **Sample** | **Read B/Read A** |
| --- | --- |
| Positive control | 40.34 |
| Negative control | 0.1 |
| C1P4 | 0.29 |

The negative should produce a ratio of < 1.0.
