# Supplementary material for: Generation of UCiPSC-derived neurospheres for cell therapy and its application
Source: Stem Cell Res Ther. 2021 Mar 18;12:188. doi: 10.1186/s13287-021-02238-4 (PMC7977190; doi:10.1186/s13287-021-02238-4)
Supplement: Supplementary file 5 — Additional file 5: Supplementary Table 2. Antibodies used for immunocytochemistry. [file 13287_2021_2238_MOESM5_ESM.docx]

| **Antibodies used for immunocytochemistry** | | | |
| --- | --- | --- | --- |
|  | **Antibody** | **Dilution** | **Company & Cat number** |
| Pluripotency Markers | Rabbit anti- NANOG | 1:200 | Cell Signaling Technology, 4903T |
|  |  |  |  |
| NSC Markers | Rabbit anti-PAX6 | 1:50 | Abcam, ab5790 |
| NSC Markers | Rabbit anti-Nestin | 1:1000 | Abcam, ab134017 |
| Neuron Markers | Rabbit anti-TUJ1 | 1:400 | Millipore, AB15708A4 |
| Neuron Markers | Rabbit anti-MAP2 | 1:500 | Millipore, AB5622 |
| Astrocyte Markers | Rabbit anti-GFAP | 1:250 | Abcam, ab33922 |
| Secondary antibodies | Alexa Fluor 488- Goat Anti-Rabbit IgG | 1:300 | Proteintech Group,  SA00006-2 |
| Secondary antibodies | Alexa Fluor594- Donkey Anti-Chicken | 1:300 | Jackson Immunoresearch,  130446 |
|  |  |  |  |
| **Primers** | | | |
| **Target** | | **Forward/Reverse primer (5′-3′)** | |
| hOCT4-F  hOCT4-R | | CCTCACTTCACTGCACTGTA  CCTCACTTCACTGCACTGTA | |
| hSOX2-F  hSOX2-R | | CCCAGCAGACTTCACATGT  CCTCCCATTTCCCTCGTTTT | |
| hNANOG-F  hNANOG-R | | TGAACCTCAGCTACAAACAG  TGAACCTCAGCTACAAACAG | |
| hGATA4-F  hGATA4-R | | CAGAAAACGGAAGCCCAA  TTGCTGGAGTTGCTGGAAG | |
| hAFP-F  hAFP-F | | ATTGGCAAAGCGAAGCTG  GCTGTGGCTGCCATTTTT | |
| hTBX1-F  hTBX1-R | | AGCGAGAAATATGCCGAGG  TTCGCGAAGGGATTGCT | |
| hPAX6-F  hPAX6-R | | TTGCTTGGGAAATCCGAG  TGCCCGTTCAACATCCTT | |
| hSOX1-F  hSOX1-R | | TTTCCCCTCGCTTTCTCA  TGCAGGCTGAATTCGGTT | |
| hNestin-F  hNestin-R | | CCACCCTGCAAAGGGAATCT  GGTGAGCTTGGGCACAAAAG | |
| hGAPDH-F  hGAPDH-R | | ACACCCACTCCTCCACCTTT  TTACTCCTTGGAGGCCATGT | |
